# Activation of the chemokine receptor 3 pathway leads to a better response to immune checkpoint inhibitors in patients with metastatic urothelial carcinoma

**DOI:** 10.1186/s12935-022-02604-z

**Published:** 2022-05-13

**Authors:** Wenqin Feng, Anqi Lin, Le Sun, Ting Wei, Haoxuan Ying, Jian Zhang, Peng Luo, Weiliang Zhu

**Affiliations:** Department of Oncology, Zhujiang Hospital, Southern Medical University, 253 Industrial Avenue, Guangzhou, 510282 Guangdong China

**Keywords:** CXCR3 pathway, Immunotherapy, Immune microenvironment, Immune checkpoint inhibitors, Urothelial cancer

## Abstract

Immune checkpoint inhibitors (ICIs) have made important breakthrough in anti-tumor therapy, however, no single biomarker can accurately predict their efficacy. Studies have found that tumor microenvironment is a key factor for determining the response to ICI therapy. Cytokine receptor 3 (C-X-C Motif Chemokine Receptor 3, CXCR3) pathway has been reported to play an important role in the migration, activation, and response of immune cells. We analyzed survival data, genomics, and clinical data from patients with metastatic urothelial carcinoma (mUC) who received ICI treatment to explore the relationship between CXCR3 pathway activation and the effectiveness of ICIs. The Cancer Genome Atlas Bladder Urothelial Carcinoma cohort and six other cohorts receiving ICI treatment were used for mechanism exploration and validation. In the ICI cohort, we performed univariate and multivariate COX analyses and discovered that patients in the CXCR3-high group were more sensitive to ICI treatment. A Kaplan–Meier analysis demonstrated that patients in the high CXCR3-high group had a better prognosis than those in the CXCR3-low group (P = 0.0001, Hazard Ratio = 0.56; 95% CI 0.42−0.75). CIBERSORT analysis found that mUC patients in the CXCR3-high group had higher levels of activated CD8+ T cells, M1 macrophages, and activated NK cells and less regulatory T cell (Treg) infiltration. Immunogenicity analysis showed the CXCR3-high group had higher tumor neoantigen burden (TNB). Our study suggests that CXCR3 pathway activation may be a novel predictive biomarker for the effectiveness of immunotherapy in mUC patients.

**Supplementary Information:**

The online version contains supplementary material available at 10.1186/s12935-022-02604-z.

## Introduction

Bladder cancer is a malignant cancer originating from the urothelium of the bladder and is one of the most common malignant tumors in the urinary system. Bladder cancer primarily includes urothelial (transitional cell) carcinoma, squamous cell carcinoma, and adenocarcinoma. Among these, urothelial carcinoma is the most common type, and accounts for more than 90% of bladder cancers [1–3]. Cisplatin-based chemotherapy is the established standard first-line therapy for metastatic urothelial carcinoma (mUC) [4–6]. However, mUC recurrence rates are high, and a large proportion of patients cannot receive cisplatin therapy for various reasons. Over the past few decades, the long-term survival rate of bladder cancer has not improved much due to the limited treatment options. The 5-year survival rate for patients with distant metastatic urothelial carcinoma was only 5% before the introduction of immune checkpoint inhibitors [4–6]. The median overall survival (OS) time with standard chemotherapy regimens was 14–15 months [7, 8]], and patients who were refractory to platinum-based chemotherapy had a median OS of approximately 7 months after receiving the second-line or another chemotherapy regimen [9]. However, several studies have reported that among mUC patients who are refractory to standard chemotherapy, those treated with ICIs have a median OS of 11–17 months [10–12], therefore, the emergence of immunotherapy has brought new hope for the treatment of mUC patients.

In 2016, the American Food and Drug Administration (FDA) approved five programmed cell death protein 1/programmed cell death ligand 1 (PD-1/PD-L1) monoclonal antibodies for second-line treatment of metastatic bladder cancer, including atezolizumab, nivolumab, durvalumab, pembrolizumab and avelumab [13]. The Keynote-045 [14] and IMvigor-210 cohort2 [15] have demonstrated that ICI treatment can extend survival time compared to traditional chemotherapy. Currently, based on the results of the KEYNOTE-052 [16], IMvigor-210 [17], KEYNOTE-361 [11], and IMvigor130 [18] studies, the FDA has approved pembrolizumab and atezolizumab as first-line treatments for mUC patients who cannot tolerate cisplatin or carboplatin therapy but are PD-L1 positive [19]. Although the overall response rate has improved recently, about 80% of patients do not benefit from ICI treatment [20]. Therefore, identifying those with better ICI responses is important for precision medical therapy [21, 22]. Biomarkers such as the tumor mutation burden (TMB), PD-L1 expression, deficient mismatch repair gene expression or microsatellite instability-high (dMMR/MSI-H), tumor-infiltrating lymphocytes, and gene expression programming (TILs/GEP) are reported to be able to predict ICI efficacy [23–27]. However, these biomarkers remain imperfect. For example, as a target of ICIs, PD-L1 expression should theoretically be a stable indicator of ICI effectiveness. However, in different types of tumors or in different parts of the same tumor, PD-L1 expression is different. Moreover, PD-L1 is inducible and its expression can change over time. Additionally, methods for detecting biomarkers across different platforms are inconsistent and controversial. These challenges make it difficult to be widely used in clinical application [28]. Some researchers have also proposed combining multiple biomarkers for prediction of ICI effectiveness, but results from this approach are not ideal [29–32]. In fact, there are correlations between biomarkers and ICI effectiveness, the search for effective and stable biomarkers is essentially an exploration into the mechanisms of ICI. Therefore, the search for clinically accessible and stable biomarkers for ICI effectiveness has a long way to go.

CXCR3 is a chemokine receptor that can be activated by the interferon-(IFN)-γ inducible ligands Chemokine (CXC motif) Ligand 9 (CXCL9), Chemokine (CXC motif) Ligand 10 (CXCL10), and Chemokine (CXC motif) Ligand 11 (CXCL11). The roles of CXCR3 in tumor progression or inhibition have been reported in recent studies [33]. Lunardi et al. found that the expression of CXCR3 in pancreatic cancer tissues is associated with tumor metastasis and poor prognosis [34].In the tumor microenvironment, it has been demonstrated that the CXCR3 signaling pathway can activate anti-tumor effector T cells and other immune cells such as NK cells, turning a “cold” tumor into a “hot” tumor and improving anti-tumor immunity. However, the mechanism of action for how the CXCR3 pathway impacts anti-tumor immunity has not yet been determined [35–37]. Unlike the PD-1/PD-L1 pathway, which plays a central role in regulating T cell exhaustion, the CXCR3 axis can promote the growth of effector T cells and killing of tumor cells, which implies that the CXCR3 axis may impact the effectiveness of tumor immunotherapy. However, only a few preclinical experiments have explored the relationship between CXCR3 signaling pathway and cancer immunotherapy [35], and due to the complexity of the immune system, immune responses may vary in different individuals and different types of tumors. Thus, the relationship between the CXCR3 axis and the response to ICIs is unclear.

To clarify the relationship between CXCR3 pathway activation and the effectiveness of ICI treatment and prognosis of mUC patients, we downloaded the clinical, genomic, and transcriptomic data from the mUC ICI and TCGA BLCA cohorts and comprehensively analyzed them using bioinformatic methods. We also explored the underlying mechanism for the relationship between CXCR3 signaling pathway and ICI effectiveness at the genomic and cellular levels.

## Material and methods

### Acquisition of public data

We downloaded ICI cohort data from the IMvigor210CoreBiologies package [38], which included genomic, transcriptomic, and clinical data from 348 mUC patients treated with anti-PD-L1 drugs. We also downloaded mutational, transcriptional, and clinical data from The Cancer Genome Atlas Bladder Cancer (TCGA-BLCA) cohort using the “TCGAbiolinks” package [39]. To validate our results, RNA-seq data and clinical data from GSE135222, GSE126044, GSE93157, GSE35640, and GSE140901 were downloaded from the Gene Expression Omnibus (GEO) database. Expression data from PRJEB23709 were obtained from a previously published study [40]. Transcripts per kilobase of exon model per million mapped reads (TPM) [41] was used to quantify RNA expression levels. A detailed workflow is shown in Additional file 1: Figure S1.

### Analysis of the CXCR3 pathway’s value for predicting treatment efficacy and prognosis of immunotherapy-treated urothelial carcinoma patients

We obtained 298 samples from the ICI cohort with both efficacy and prognostic information. We then collected gene sets associated with the CXCR3 pathway from the Molecular Signatures Database (MSigDB) [42]. After converting RNA-seq raw counts to TPM (Transcripts Per Million) matrices, we performed single-sample gene set enrichment analysis (ssGSEA) [43] using the GSVA package [44]. The ssGSEA score was used to quantify activation levels of the CXCR3 pathway, and the mUC patients were divided into CXCR3-high and -low groups according to the median ssGSEA value. To assess the capacity of the CXCR3 pathway to predict ICI effectiveness, we performed univariate Cox regression analysis on clinical indicators and CXCR3 scores in the ICI cohort. After excluding indicators with multicollinearity, potential statistical significance indicators (p < 0.05) were included in the multivariate Cox regression model. Kaplan–Meier survival analysis and log-rank test were used to assess survival differences between the two groups.

In addition, we assessed the relationship between CXCR3 pathway activation and ICI efficacy in another six cohorts (GSE135222, GSE126044, GSE93157, GSE35640, GSE140901, PRJEB23709). We used ROC (receiver operating characteristic) curve analysis to calculate the sensitivity and specificity of the CXCR3 ssGSEA score from the validation cohort. We then divided these patients into CXCR3-high and low groups according to the optimal cut-off point calculated by the "surv_cutpoint" function of the "surviminer" R package [45] and compared the efficacy of ICI treatment between the CXCR3-high and -low groups.

### Analysis of mutation and immunogenicity data

To elucidate the relationship between the CXCR3 pathway activation and genomic mutation characteristics, immunogenicity of UC patients, we compared the differences in the mutation rates of the top 20 driver genes between the CXCR3-high and -low groups in the ICI and TCGA cohorts. The driver gene mutation panorama was visualized by the “ComplexHeatmap” R package [46].

We also compared differences in the TMB, TNB, and DNA repair and DNA damage response (DDR) pathway mutation counts between the CXCR3-high and CXCR3-low groups in the ICI and TCGA cohorts. The DDR pathway gene set was downloaded from the Molecular Signature Database (MSigDB) [42]. TMB and TNB data were obtained from the clinical information from the corresponding cohort [38, 39]. In addition, MANTIS, a score that predicts a patient’s MSI status, was downloaded from cBioPortal [47] for the TCGA-BLCA cohort. TMB was defined as the number of somatic, coding, base substitution, and indel mutations per megabase of genome examined [48]. The calculation method for TNB was obtained from previous literature [49]. Mutual exclusion analysis of driver mutant genes in the above cohort was visualized by the "Maftools" R package [50].

### TME analysis and pathway enrichment analysis

To reveal the underlying mechanisms for how the CXCR3 pathway impacts immunotherapy, we used the CIBERSORT method (1000 iterations; parameters: default) [51] to compare the proportions of 22 types of immune cells between the CXCR3-high and CXCR3-low groups. We used the DESeq2 [52] package to perform differential gene analysis between the CXCR3-high and CXCR3-low groups both in the ICI cohort and the TCGA-BLCA cohort. We then compared differences in immune-related gene expression published by Thorsson et al. [49]. After differential gene analysis, we used the “clusterProfiler” R package [53] to perform Gene Set Enrichment Analysis (GSEA) [43]. P values less than 0.05 were considered statistically significant, and Gene Ontology (GO), REACTOME and Kyoto Encyclopedia of Genes and Genomes (KEGG) terms were obtained from the MSigDB database [42].

### Analysis of drug sensitivity

To discover drugs that could improve the efficacy of ICI treatment, we conducted drug analysis in both the CLUE (https://clue.io/) and GDSC (Genomics of Drug Sensitivity in Cancer) databases. First, we performed differential gene analysis for the CXCR3-high and -low groups in the ICI and TCGA BLCA cohorts and converted gene IDs to GPL96 format. Next, we uploaded the top 500 differentially up- and down-regulated genes into the CLUE database for cMap (Connectivity Map) analysis [54]. In addition, we used the R package “pRRophetic” [55] to conduct drug sensitivity analysis of 138 small and medium molecule drugs from the GDSC database.

### Immunohistochemistry

Clinical information and corresponding pathological tissues before immunotherapy were retrospectively collected from two patients who were diagnosed with primary urothelial bladder carcinoma at Zhujiang Hospital, and the patients were divided into responder and non-responder group for ICI treatment according to Response evaluation criteria in solid tumors (RECIST) criteria. The study was approved by the Ethics Committee of Zhujiang Hospital, Guangdong Province. The obtained tissues were formalin-fixed, dehydrated and made transparent, paraffin-embedded, and cut into 3 μm thick sections using a microtome. The slides were baked in a constant temperature oven at 60 °C for 1 h, immediately placed into xylene for dewaxing, and soaked twice. The dewaxed slides were rehydrated in alcohol with a range of gradually decreasing concentrations. After autoclaving at 115 °C for 5 min for antigen repair in citrate buffer (pH 6.2), the endogenous peroxidase activity solution was quenched with 3% H_2_O_2_ for 15 min. Slides were then blocked with normal goat serum for 15 min and incubated with anti-CXCR3 (1:200 dilution), anti-CXCL9 (1:100), anti-CXCL10 (1:50) primary antibody overnight at 4˚C. All antibodies were manufactured by Proteintech company. The slides were then treated with goat anti-mouse secondary antibody for 15 min at room temperature. The slides were stained by the DAB visualization kit (SP Rabbit HRP Kit (DAB); CW2035S, China) and restained with hematoxylin. Images were acquired by slide scanner (NanoZoomer 2.0-HT; HAMMATSU, NIKON, Japan) and were semi-quantified with semi-quantitative scores. Besides, we used ImageJ software [56] to analyze the percentage contribution of positive area and visualize the results with GraphPad Prism Version8.4.0.

### Statistical analysis

Univariate and multivariate Cox regression models were used to determine the predictive value of the CXCR3 pathway score in mUC patients treated with ICI. Differences in gene mutation rates and response proportions in ICI treated patients between the CXCR3-high and -low groups were assessed by Fisher's exact test. Overall survival time was estimated by the Kaplan–Meier method, and the OS difference between the two groups was estimated by the log-rank test. Wilcoxon's test was used to estimate differences in CXCR3 pathway scores, immune cell proportions, immune-related genes, and drug sensitivity between groups. The Kruskal–Wallis test was used to compare the differences in PD-L1 expression in tumor cells (TC) and immune cells (IC) between groups, it was also used to compare the differences of CXCR3 pathway scores between groups. Chi-Square test was used to compare the differences of tumor stages in patients in CXCR3-high and low groups. Spearman correlation analysis was used to test the correlation between CXCR3 scores and tumor mutational burden (TMB) and tumor neoantigen burden (TNB). Quantification results of immunohistochemistry-stained sections in CXCR3 pathway were compared with t-test. R software (version 4.0) was used for statistical analysis, and p < 0.05 was considered statistically significant.

## Results

### CXCR3 pathway score can be used as a predictor of response to Immunotherapy in mUC patients.

**Fig. 1:**
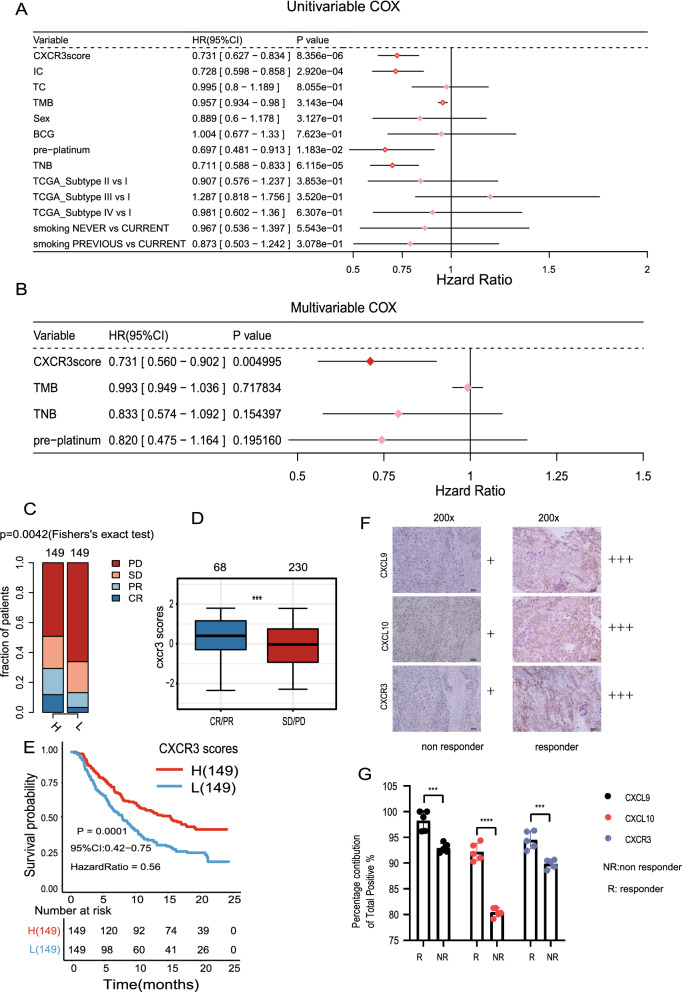
Activation of the CXCR3 pathway indicates better efficacy of ICI in mUC patients. **A** The results of univariate Cox regression analysis are shown in Forest plot. The indicators with p < 0.05 are CXCR3 score, IC level, TMB, TNB and platinum therapy. **B** The Forest plot visualizes the results of multivariate Cox regression analysis. Results showed that CXCR3 score is a potential prognostic factor of ICI in UC patients. HR indicates mUC patients have a favorable prognosis (HR < 1) or a poor prognosis (HR > 1). **C** The proportion of mUC patients with different responses to ICI between CXCR3-high and CXCR3-low patients in the ICI cohort. CR: complete response; PR: partial response; PD: progressive disease; SD: stable disease. **D** Differences in CXCR3 pathway activation between CR/PR and SD/PD patients. The asterisks above the box plot indicate the range of p values. “.”: p < 0.1; “*”: p < 0.05; “**”: p < 0.01; “***”: p < 0.001. **E** Kaplan-Meier survival curves for OS in CXCR3-high (n = 149) and CXCR3-low(n = 149) patients in the ICI cohort. **F** the expression of CXCR3 pathway core proteins in non-responder and responder UC patients treated with ICI therapy. Staining for immunoreactivity was assessed by semi-quantitative scoring. − : 0%; + : < 30%; +  + : 30–60%; +  +  + : > 60% of immunoreactive cells throughout the tissue. ICI = immune checkpoint inhibitors. **G** Comparative analysis of immunohistochemical staining intensity determined by ImageJ. The results were evaluated by t-test. Statistically significant results are marked by asterisk (*) directly in graph. * P < 0.05, ** P < 0.01, *** P < 0.001

To explore the effect of CXCR3 pathway activation on response to immunotherapy in mUC patients, we selected clinical variables related to ICI treatment and CXCR3 pathway score and performed univariate Cox regression analysis (Fig. 1A). Simultaneously, after eliminating the influence of multicollinearity factors, variables with statistical and clinical significance and p < 0.05 were analyzed by multivariate COX regression analysis. The multivariate Cox regression analysis found that the CXCR3 score (HR = 0.731(95%CI 0.560–0.902), p = 0.004) was independent of TNB (HR = 0.833 (95% CI 0.5740–1.092), p = 0.154), TMB (HR = 0.993 (95% CI 0.949–1.036), p = 0.717) and pre-platinum (HR = 0.820 (95% CI 0.475–1.164), p = 0.195) and was a predictor of prognosis for mUC patients with ICI therapy (Fig. 1B). This indicated that greater CXCR3 pathway activation predicted a better response to immunotherapy in mUC patients.

To evaluate whether CXCR3 pathway activation can predict the prognosis of mUC patients and the efficacy of ICI treatment, we divided the patients into responders (complete or partial response, CR or PR) and non-responders (stable or progressive disease, SD or PD) according to their response to treatment. The responders had higher CXCR3 pathway activation levels than the non-responders (Wilcoxon test, p < 0.001, Fig. 1D). According to the median value of CXCR3 pathway ssGSEA scores, we grouped the patients into CXCR3-high and CXCR3-low groups, which represent the high and low activation level of CXCR3 pathway. The results showed there were more ICI responders in the CXCR3-high group than in the CXCR3-low group (two-tailed Fisher's exact test, p = 0.0042, Fig. 1C). We then performed survival analysis on the ICI cohort and found that patients with high CXCR3 pathway activation levels had better prognoses than those with low CXCR3 pathway activation levels (log-rank test, HR 0.56 [95% CI 0.42–0.75], p = 1e-04, Fig. 1E). A similar survival analysis was performed on the TCGA-BLCA cohort, and the survival trend was similar to that for the ICI cohort but did not reach statistical significance (log-rank test, HR 0.85 [95% CI 0.63–1.14], p = 0.281, Additional file 4: Figure S4E). These results may demonstrate that the predictive capacity of CXCR3 pathway activation is more reliable for mUC patients treated with ICI therapy.

### Analysis of gene mutations and clinical features

**Fig. 2:**
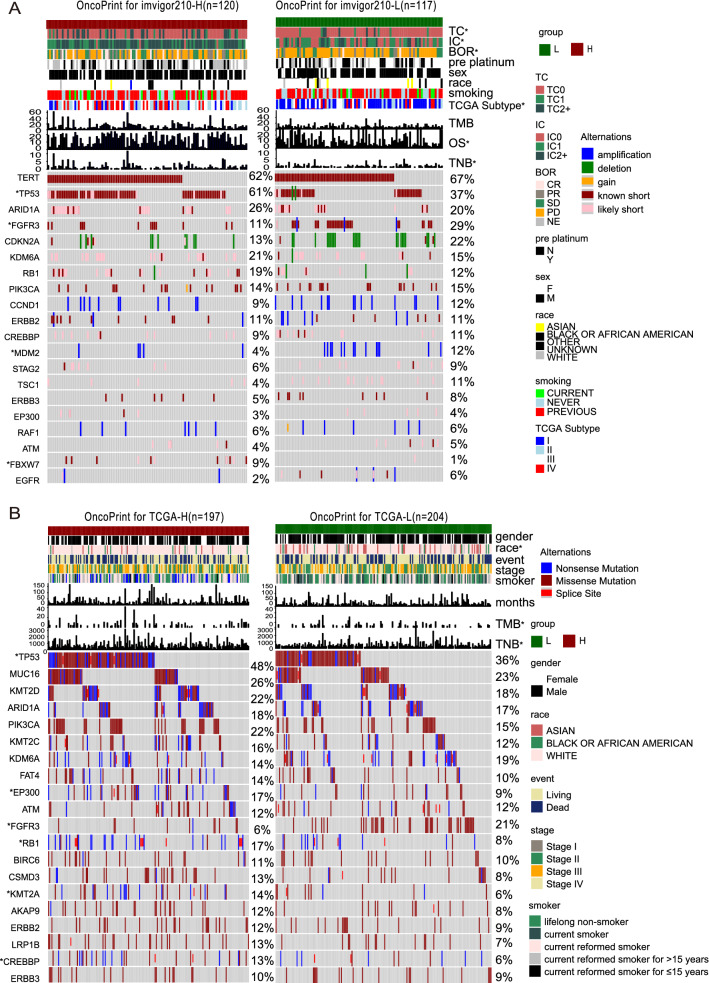
Genomic profiles of UC patients in the ICI-treated cohort **(A)** and TCGA-BLCA cohort **(B)**. The figure mainly shows the top 20 driver genes with the highest mutation rates, and the right bar plot indicates the mutation rate of each driver gene. Genes mutated significantly between CXCR3-high and CXCR3-low patients are labeled with asterisks after their name. The bar plot visualizes the differences in corresponding clinical information

To explore the potential mechanism for how CXCR3 pathway activation impacts the efficacy of ICIs, we investigated the relationship between CXCR3 pathway activation and genomic alterations in mUC patients (Fig. 2). The gene mutation landscape showed the alteration types and frequencies of the top 20 driver genes in the ICI and TCGA cohorts. We observed a total of eight genes with significant differences in mutation frequencies between CXCR3-high and CXCR3-low patients, which were TP53 (61% vs 37%, p < 0.05), FGFR3 (11% vs 29%, p < 0.05), MDM2 (4% vs 12%, p < 0.05), and FBXW7 (9% vs 1%, p < 0.05) in the ICI cohort; and TP53 (48% vs 36%, p < 0.05), EP300 (17% vs 9%, p < 0.05), FGFR3 (6% vs 21%, p < 0.05), RB1 (17% vs 8%, p < 0.05), KMT2A (14% vs 6%, p < 0.05), and CREBPP (13% vs 6%, p < 0.05) in the TCGA BLCA cohort. Detailed results for gene mutation frequency are shown in Additional file 7: Table S1.

We also compared differences in clinical variables among patients with different levels of CXCR3 pathway activation. We noticed that patients in the two cohorts with higher levels of CXCR3 pathway activation had higher tumor neoantigen burdens (Fig. 2A, B , p < 0.05). We also found statistically significant differences in the expression of PDL-1 in tumor cells (TC) and immune cells (IC) between groups with different CXCR3 pathway activation levels (Kruskal–Wallis test, p < 0.001, Additional file 4: Figure S4A, B). TC and IC represent the expression of PD-L1 of tumor cells and immune cells (TC0/IC0 indicates PD-L1 level < 1%, TC1/IC1 indicates PD-L1 level 1–5%, and TC2 + /IC2 + indicates PD-L1 level > 5%). Smoking has previously been reported to affect the prognosis of bladder cancer patients [57], but there were no differences of CXCR3 pathway activation between the different smoking status groups (Kruskal–Wallis test, *p* > 0.05, Additional file 4: Figure S4D). Interestingly, in the TCGA-BLCA cohort, ethnicity was significantly different between the CXCR3-high and CXCR3-low groups. In addition, the tumor stages of patients in CXCR3-high and low groups are significantly different in ICI cohort (Chi-Square test, p < 0.001,, Additional file 4: Figure S4C), CXCR3-high group seems to have more late-stage patients but there are no differences of tumors stages between CXCR3-high and low groups in TCGA cohort (Chi-Square test, p = 0.5, Additional file 4: Figure S4F). Accordingly, patients with advanced tumor stage have higher CXCR3 scores in ICI cohort (Kruskal–Wallis test, p < 0.001, Additional file 4 Figure S4G). But there were no significant differences of CXCR3 scores between different stages in TCGA cohort (Kruskal–Wallis test, p > 0.05, Additional file 4: Figure S4H).

### Analysis of immune microenvironment

**Fig. 3:**
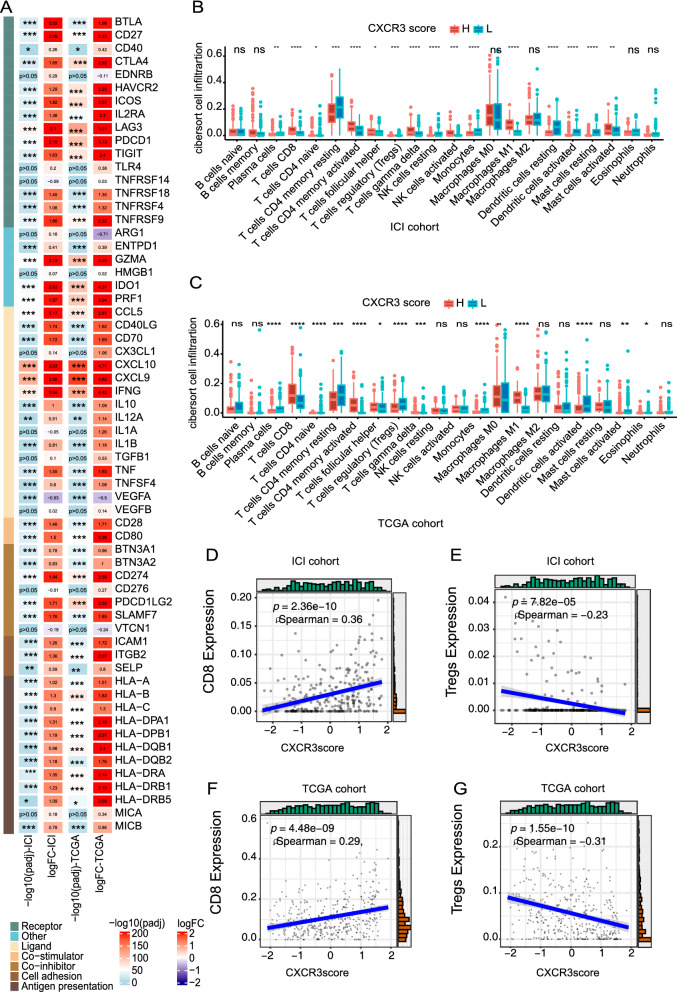
Activation of the CXCR3 pathway affects the immune microenvironment of mUC. **A** The Heatmap shows the expression levels of immune-related genes between CXCR3-high and CXCR3-low patients. The color in the first column of the heatmap represents the immune functions of the genes. The second and third columns represent the p-value and logFC analysis of the differential gene expression analysis in the ICI cohort, while the fourth and fifth are representatives of the TCGA-BLCA cohort. The color represents the size of logFC shown in the middle of the rectangles. LogFC > 0 means that the genes are highly expressed in CXCR3-high patients, while logFC < 0 is the opposite. **B** The box plot shows the differences in 22 immune cells between CXCR3-high and CXCR3-low groups according to the CIBERSORT analysis results from the ICI cohort. **C** The box plot shows the difference in 22 immune cells between CXCR3-high and CXCR3-low groups according to the CIBERSORT analysis results from theTCGA-BLCA cohort. **D** The correlation between CXCR3 pathway activation and the proportion of CD8+ T cells in the ICI cohort. **E** The correlation between CXCR3 pathway activation and the proportion of Tregs in the ICI cohort. **F** The correlation between CXCR3 pathway activation and the proportion of CD8+ T cells in the TCGA-BLCA cohort. **G** The correlation between CXCR3 pathway activation and the proportion of Tregs in the TCGA-BLCA cohort

To compare differences in the immune microenvironment for different CXCR3 pathway activation levels, we analyzed immune-related genes, immune cell infiltrations, and immunogenicity of patients with different CXCR3 pathway activation levels. Immunotherapy targets immune checkpoints, and therefore, expression of immune checkpoint genes is important for response to ICIs. In the ICI and TCGA cohorts, we found that expression of LAG3, PDCD1, and PD-L1 (CD274) was significantly elevated in the CXCR3-high patients; however, Vascular Endothelial Growth Factor A (VEGFA), a molecule that promotes tumor angiogenesis, was significantly downregulated in CXCR3-high patients (all p < 0.05; Fig. 3A). Other immune-related genes, such as cytotoxicity markers (GZMB) and cytokine-related genes (e.g. IFNG), were significantly upregulated in the CXCR3-high group (all p < 0.05). Infiltration of effector immune cells, such as CD8+ and CD4+ T cells and M1 macrophages, was higher in the CXCR3-high group (Fig. 3B, C), whereas Tregs and activated dendritic cell infiltration were higher in the CXCR3-low group. Further correlation analysis indicated that CD8+ T cell infiltration was positively correlated with CXCR3 pathway activation (ICI cohort: p < 0.001, r = 0.36; TCGA cohort: p < 0.001, r = 0.29, Fig. 3D, F), but Tregs infiltration was negatively correlated with CXCR3 pathway activation (ICI cohort: p < 0.001, r = − 0.23; TCGA cohort: p < 0.001, r = − 0.31, Fig. 3E, G).

Gene Set Enrichment Analysis (GSEA) was used to detect crosstalk between the CXCR3 pathway and other signaling pathways to influence the tumor microenvironment. We discovered that immune activation related pathways (such as adaptive immune response, T cell activation, and antigen processing and presentation) were significantly enriched in the CXCR3-high patients, while lipid metabolism and glucose metabolism signaling pathways were enriched in CXCR3-low patients (Additional file 5: Figure S5, Additional file 6: Figure S6).

**Fig. 4:**
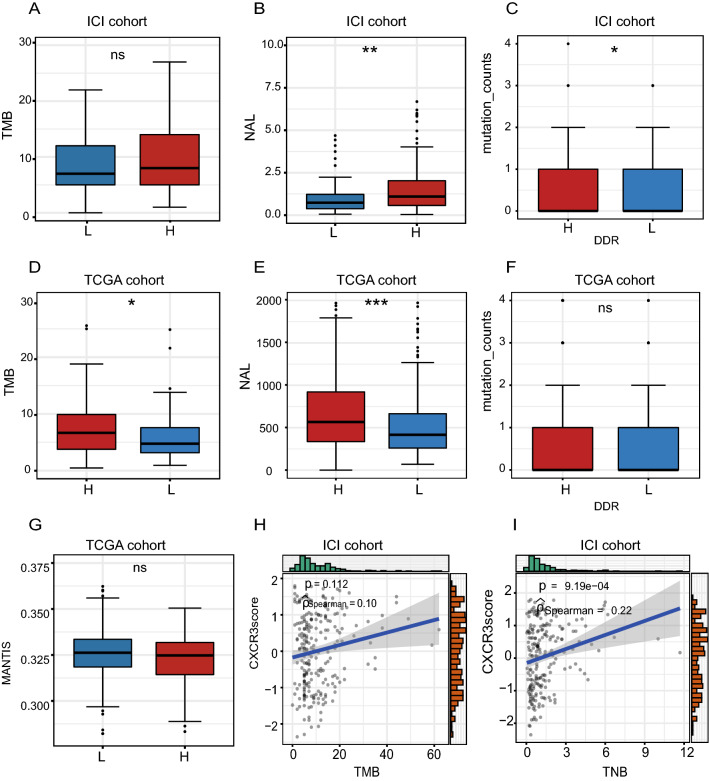
The relationship between the CXCR3 pathway and tumor immunogenicity **A** Comparison of TMB between the CXCR3-high and CXCR3-low groups in the ICI cohort. **B** Comparison of NAL between the CXCR3-high and CXCR3-low groups in the ICI cohort. **C** Comparison of DDR mutations between the CXCR3-high and CXCR3-low groups in the ICI cohort. **D** Comparison of TMB between the CXCR3-high and CXCR3-low groups in the TCGA cohort. **E** Comparison of NAL between the CXCR3-high and CXCR3-low groups in the TCGA cohort. **F** Comparison of DDR mutations between the CXCR3-high and CXCR3-low groups in the TCGA cohort. **G** Comparison of MANTIS scores between the CXCR3-high and CXCR3-low groups in the TCGA cohort. **H** The correlation between CXCR3 pathway activation and TMB in the ICI cohort. **I** The correlation between CXCR3 pathway activation and TNB in the ICI cohort

To elucidate the effect of the CXCR3 pathway on tumor immunogenicity, we compared differences in TNB, TMB, and DDR-related pathway mutation status between the CXCR3-high and CXCR3-low groups. For both the ICI and TCGA cohorts, the TMB was higher in CXCR3-high patients than in CXCR3-low patients, but this difference was only statistically significant for the TCGA cohort (ICI cohort: p > 0.05, TCGA cohort: p < 0.05, Fig. 4A, D). Similarly, in the ICI cohort, the mutation counts of the DDR-related pathways were significantly higher in CXCR3-high group. The TCGA-cohort showed the same trend but without statistical significance (ICI cohort: p < 0.05, TCGA cohort: p > 0.05, Fig. 4C, F). In both cohorts, TNB was elevated in CXCR3-high patients compared to CXCR3-low patients (ICI cohort: p < 0.001, TCGA cohort: p < 0.0001, Fig. 4B, E). The subsequent correlation analysis of TMB, TNB and CXCR3 pathway activation in the ICI cohort showed that both TMB and TNB were positively correlated with CXCR3 score (ICI cohort: p = 0.112, rSpearman = 0.1; p = 9.19e-04, rSpearman = 0.22; Fig. 4H,I). The MANTIS score was used to evaluate the microsatellite instability status (MSI) of patients, The higher the score is, the microsatellite instability status is closer to MSI-H. We found that in the TCGA cohort, MANTIS scores were higher in CXCR3-high patients than in TCGA-low patients, but this difference was not statistically significant (TCGA cohort: p > 0.05, Fig. 4G).

### Analysis of drug sensitivity

Drug sensitivity analysis can help to transform findings from research into clinical application by identifying potential drug treatment options for future application. We used the Genomics of Drug Sensitivity in Cancer (GDSC) database, the CLUE database, and the RNA transcriptome data from two bladder cancer cohorts to perform drug sensitivity analysis. Mechanism of action (MoA) analysis was used to summarize and screen the drugs’ potential mechanisms of action.

**Fig. 5:**
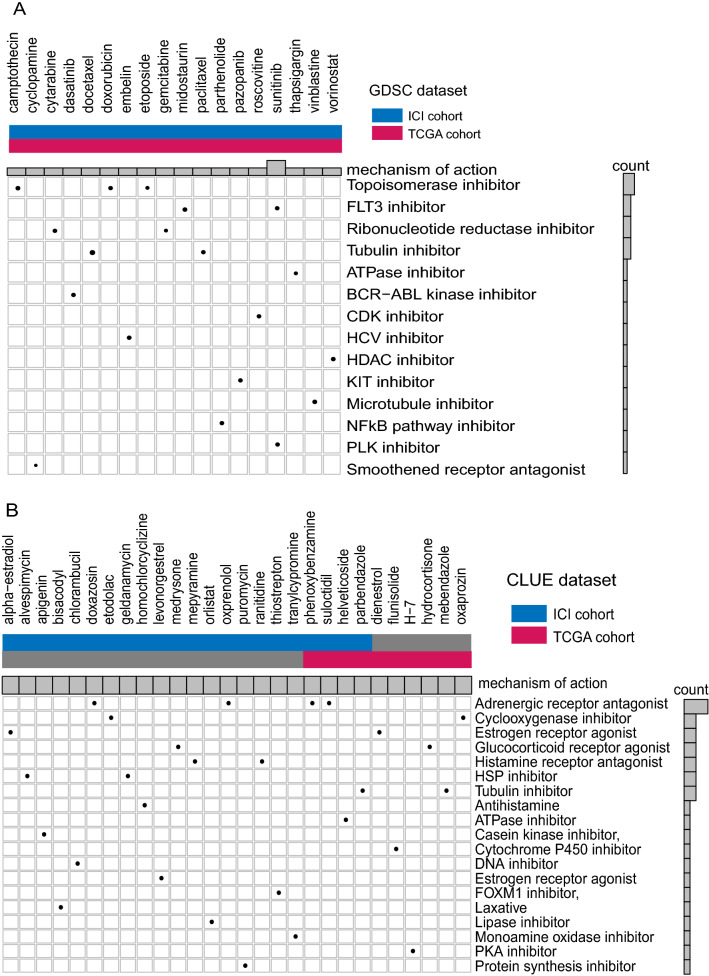
Results of drug sensitivity analysis from the GDSC database and the CMAP database. **A** The molecular mechanisms of small molecule drugs according to GDSC results. **B** The molecular mechanisms of small molecule drugs according to the CMAP results

First, we used the pRRophetic algorithm, the GDSC database, and the gene expression profiles from the ICI and TCGA cohorts to construct a ridge regression model to predict the IC50 values ​​of 138 small and medium molecule drugs. We selected 18 drugs with 14 different mechanisms of action from the ICI and TCGA cohorts (Fig. 5A). We then performed cMap analysis on the differentially expressed genes from the two cohorts and screened 28 targeted drugs according to scores p < 0.05 and ES > 0 in the CLUE database (Fig. 5B). This identified drugs that altered the mRNA profiles of cell lines to more closely resemble those of the CXCR3-high patients. These findings suggested that mUC patients were more sensitive to ICI treatment when treated with the identified drugs, which provides useful insight for ICI combination therapy.

MoA analysis revealed the molecular mechanisms of action for the drugs identified in our screen. We identified four tubulin inhibitors [58] (docetaxel, paclitaxel, parbendazole, mebendazole) and two ATPase inhibitors [58, 59] (helveticoside, thapsigargin) from the CLUE database and the GDSC database. Some studies have revealed the anti-tumor mechanisms of the above drugs. We speculate that these drugs may enhance the response to ICI therapy, but further in vitro and in vivo studies are needed to confirm this hypothesis. Detailed findings from this analysis are shown in Additional file 8: Table S2.

### CXCR3 pathway activation is also a predictor of response to immunotherapy in patients with other cancers

Finally, we validated our findings on the relationship between CXCR3 pathway activation and ICI effectiveness in cohorts of other types of cancer. We found that CXCR3 pathway activation can not only predict the effectiveness of ICIs in patients with metastatic urothelial carcinoma, but it can also predict the effectiveness of immunotherapy in patients with melanoma, non-small cell lung cancer, and liver cancer. We analyzed CXCR3 pathway ssGSEA scores in patients with liver cancer, melanoma, and non-small cell lung cancer who received immunotherapy and found that CXCR3 pathway scores in the responder groups were significantly higher than those in the non-responder groups (Wilcoxon's test, GSE35640, p = 0.006; GSE14091, p = 0.0034; GSE93157, p = 0.095; GSE126044, p = 0.027; GSE135222, p = 0.084; PRJEB23709, p = 8.8e-06; Additional file 2: Figure S2G–L). To achieve the best validation effect, we used ROC curve analysis to obtain the optimal threshold (Additional file 3: Figure S3) and divided patients into CXCR3-high and CXCR3-low groups according to the optimal threshold. We found that there were more responders in the CXCR3-high group than in the CXCR3-low group (Fisher's exact test, GSE35640, p = 0.0061; GSE14091, p = 0.034; GSE93157, p = 0.04; GSE126044, p = 0.026; GSE135222, p = 0.033; PRJEB23709, p = 5e-06; Additional file 2 Figure S2A–F). These conclusions validate our findings from mUC patients and suggest an association between CXCR3 pathway activation and response to immunotherapy.

### Immunohistochemistry

To investigate the clinical significance of CXCR3 pathway activation and response to immunotherapy in patients with urothelial bladder carcinoma, we performed immunohistochemical analysis on two urothelial carcinoma patients treated with ICI therapy at Zhujiang hospital. Immunohistochemistry showed that three core proteins associated with the CXCR3 pathway, CXCR3, CXCL9, CXCL10, and were more highly expressed in cancer tissues from the immunotherapy-responder group than in tissues from the immunotherapy non-responder group (n = 1 per group) (Fig. 1F).

We used Image J software to quantify the immunochemistry-stained results of three CXCR3 pathway core proteins, and use GraphPad Prism to visualize the results (T-test, CXCL9,p = 0.0005;CXCL10,p = 0.0001, CXCR3,p = 0.0007; Fig. 1G**)**. In conclusion, from analyzing publicly available datasets and our own clinical immunohistochemistry specimens, we conclude that higher levels of CXCR3 pathway activation are correlated with a better response to immunotherapy in patients with urothelial bladder carcinoma.

## Discussion

In our study, by analyzing clinical, transcriptomic, and genomic data from the ICI and TCGA-BLCA cohorts, we found that mUC patients with higher CXCR3 pathway activation levels responded better to immunotherapy and had longer overall survival (OS) time than patients with lower CXCR3 pathway activation levels. This suggests that CXCR3 pathway activation can be used as a biomarker for predicting the efficacy of ICI therapy in mUC patients (Fig. 1A–F), or that higher CXCR3 pathway activation can improve ICI treatment outcomes and the prognosis of mUC patients.

Tumor immunogenicity is defined as the ability of a tumor to induce an immune response that can prevent its growth, and the complex mechanisms for tumor immunogenicity are still a matter of investigation. Currently, tumor immunogenicity is represented by metrics including TMB, TNB, and DDR-related pathway mutation status, and some studies have reported these metrics as markers of response to immunotherapy [23, 60–63]. Our analysis of immunogenicity showed that the TNB in CXCR3-high patients was significantly higher than that in CXCR3-low patients, and there was a significant positive correlation between CXCR3 pathway activation level and TNB, which may explain why mUC patients with high CXCR3 pathway activation have better prognosis and responses to ICIs. TNB, which refers to the number of neoantigens per megabase in a genomic region, has been determined a predictive biomarker for response to immunotherapy by multiple studies [62, 64–67]. A high TNB can promote tumor recognition and T cell activation, which increases the proportion of tumor-infiltrating lymphocytes (TILs) and improves anti-tumor immunity [38, 68–70].

It has been reported that TNB is positively correlated with tumor mutational burden (TMB) and mutations in the DDR pathways, which maintain the stability of the human genome [66, 71–73]. The underlying mechanism for this correlation is that the TMB can generate neoantigens which promote tumor immunogenicity, and DDR pathway mutations lead to an accumulation of DNA damage, including somatic mutation of exons, resulting in the production of mutated proteins that are neoantigens [71, 72]. Neoantigens can activate antitumor immune responses, including CD8+ T cell infiltration and cytolytic activity, which are correlated closely with response to ICI therapy [65–67]. We compared differences in TMB and DDR pathway mutations in the CXCR3-high and -low groups in two cohorts and found that patients in the CXCR3-high group had higher TMB and more DDR pathway mutations than patients in the CXCR3-low group, but these differences were not statistically significant, likely due to limited sample size. In addition to tumor immunogenicity, the tumor immune microenvironment consisting of tumor cells, immune cells, and other stromal cells plays an important role in the response to ICI treatment. Competition between immune cells and tumor cells determines the efficacy of immunotherapy [74–76]. We performed TME analysis with the CIBERSORT algorithm and found that there were more activated CD4+ T cells, CD8+ T cells, and M1 macrophages and fewer Tregs in the TME of CXCR3-high patients than in CXCR3-low patients. This discovery indicates a positive correlation between CXCR3 pathway activation and anti-tumor immunity [77–80]. Consistent with this finding, our GSEA analysis found that immune and anti-tumor related signaling pathways, such as Toll-like receptors, Fc-γ receptors, and Major Histocompatibility Complex-I and -II (MHC-I and MHC-II) signaling pathways were upregulated in the CXCR3-high group compared to the CXCR3-low group. These results also help to explain the differences in immune cells infiltrations between the CXCR3-high and -low groups.

In addition, GSEA analysis showed that lipid metabolism pathways were downregulated in CXCR3-high patients (Additional file 5: Figure S5, Additional file **6**: Figure S6).

It has been reported that lipid metabolism is crucial for the immunosuppressive TME. The accumulation of fatty acids (FAs) can impair the cytotoxicity of effector T cells, while Tregs, which are immunosuppressive CD4+ T cells, rely on fatty acid oxidation (FAO). Furthermore, non-glycolytic, high lipid metabolism impairs the antigen-presenting capacity of dendritic cells (DCs) [81]. We analyzed gene mutation frequencies in the two groups, and consistent with our previous findings, we observed that the frequency of MDM2 (MDM2 Proto-Oncogene) mutations was higher in CXCR3-low patients than in CXCR3-high patients. MDM2 is a negative regulator of the tumor suppressor gene p53 and can act as a heteromeric complex to inhibit p53’s function [82]. It has been reported that MDM2 inhibitors can upregulate various lipids including phosphatidylcholine (PC), phosphatidylethanolamine (PE), and free fatty acid (FA), thereby inhibiting the staged metabolism of fat and resulting in increased fat synthesis and impaired lipid catabolism [83–85]. These findings help to explain the differences in immune cells infiltrations in the TMEs of CXCR3-high and low patients.

Some studies have reported that ICI combination therapy is superior to ICI monotherapy. To discover drugs with potential use in ICI combination therapy, we conducted drug sensitivity analysis. We found that tubulin inhibitors can improve the efficacy of ICIs, which is consistent with previously reported findings. It has been demonstrated that the combined use of tubulin inhibitors and ICIs was successful for the treatment of non-small cell lung cancer [86–88], because tubulin inhibitors enhanced CD4 + and CD8+ T cell activity [89], inhibited Tregs [90], induced dendritic cell maturation, and increased M1 macrophages [91, 92]. Our study suggests that tubulin inhibitors may “heat” the tumor immune microenvironment by activating the CXCR3 pathway, thereby improving the efficacy of ICI therapy. Further validation is needed to confirm the mechanism by which tubulin inhibitors increase the efficacy of ICIs.

Our study had several limitations. First, due to limited genomic and clinical survival data on bladder cancer patients treated with immunotherapy, we used immunotherapy cohorts from other cancers for validation. Second, our study did not find a stable and consistent threshold to determine CXCR3 pathway activation levels. In the present study, we used the median CXCR3 pathway activation values for the ICI and TCGA cohorts to categorize patients as CXCR3-high or CXCR3-low; however, different cancers may have different levels of CXCR3 activation, which makes this threshold difficult to apply in the clinical setting. Third, our analysis is to evaluate the average CXCR3 activation levels of cancer patients, but CXCR3 activation in different regions of a tumor may be different due to tumor heterogeneity. Further study needs to be done to explore the tumor heterogeneity’s impact on CXCR3 pathway activation levels. Four, our analysis was mainly limited to bioinformatics analysis. In future studies, we hope to perform animal and cell line experiments and even analyze a larger clinical cohort to determine the influence of the CXCR3 pathway on response to ICI treatment.

## Conclusions

Our study demonstrated that mUC patients with higher CXCR3 pathway activation levels had longer overall survival time and better treatment outcomes after receiving immunotherapy. In addition, we found that when the CXCR3 pathway is highly activated, there are more activated effector immune cells in the TME and greater upregulation of immune-related genes. Therefore, CXCR3 pathway activation can be used as a predictive biomarker to guide bladder cancer patients before receiving immunotherapy. Our findings should be confirmed by prospective clinical studies and molecular mechanistic experiments.

## Supplementary Information

**Additional file 1: Figure S1.** **A**–**C** Flow chart of the data processing of this study.

**Additional file 2: Figure S2.** Validation of relationship between CXCR3 pathway activation and efficacy of ICIs in cancer patients. **A**–**F** The differences in the proportions of mUC patients with different responses to ICIs between CXCR3-high and CXCR3-low patients in the other six ICI cohorts. Light blue color represents the responders; dark blue color represents the non-responders. **G**–**L** The differences in activation of the CXCR3 pathway in CXCR3-high and CXCR3-low patients from the other six ICI cohorts. *NR* no response, *R* response.

**Additional file 3: Figure S3.** **A**–**F** Receiver operating characteristic (ROC) curve for the predictive value of ssGSEA scores of CXCR3 pathway in the other six validation cohorts. 

**Additional file 4: Figure S4.** **A** Differences in CXCR3 pathway activation between patients with different immune cells’PD-L1 expression in the ICI cohort. **B** Differences in CXCR3 pathway activation between patients with different tumor cells’PD-L1 expression in the ICI cohort. **C** Differences in tumor stages in patients with different levels of CXCR3 pathway activation in the ICI cohort. **D** Differences in patients’ smoking history for different levels of CXCR3 pathway activation in the ICI cohort. **E** Kaplan-Meier survival curves for OS in CXCR3-high (n =202) and CXCR3-low (n =204) patients in TCGA BLCA cohort. **F** Differences in tumor stages in patients with different levels of CXCR3 pathway activation in the TCGA BLCA cohort. **G** Differences in CXCR3 pathway activation levels between patients with different tumor stages in ICI cohort. **H** Differences in CXCR3 pathway activation levels between patients with different tumor stages in TCGA BLCA cohort.

**Additional file 5: Figure S5.** Histogram showing that the ssGSEA score of the immune-related and lipid metabolism-related signaling pathways set was different in CXCR3-high and CXCR3-low patients from TCGA BLCA cohort (logFC < 0, p < 0.05). ES > 0 means that the corresponding pathway is significantly enriched in CXCR3-high patients, while ES <0 means the opposite.

**Additional file 6: Figure S6.** Histogram showing that the ssGSEA score of the immune-related and lipid metabolism-related signaling pathways set was different in CXCR3-high and CXCR3-low patients from ICI cohort (logFC < 0, p < 0.05). ES > 0 means that the corresponding pathway is significantly enriched in CXCR3-high patients while ES < 0 means the opposite.

**Additional file 7: Table S1.** Results of gene mutation frequency analysis of the ICI cohort and the TCGA BLCA cohort.

**Additional file 8: Table S2.** Results of drug sensitivity analysis from the GDSC database for the ICI cohort and the TCGA BLCA cohort.

## Data Availability

TCGA-BLCA can be downloaded from UCSC Xeno (https://xena.ucsc.edu/public). GSE135222, GSE126044, GSE93157, GSE35640, and GSE140901 are accessible in Gene Expression Omnibus (https://www.ncbi.nlm.nih.gov/geo). PRJEB23709 and other datasets used and/or analyzed during the current study are available from the corresponding author on reasonable request.
