# Supplementary figures and images for: Activation of the chemokine receptor 3 pathway leads to a better response to immune checkpoint inhibitors in patients with metastatic urothelial carcinoma
Source: Cancer Cell Int. 2022 May 13;22:186. doi: 10.1186/s12935-022-02604-z (PMC9107140; doi:10.1186/s12935-022-02604-z)

A

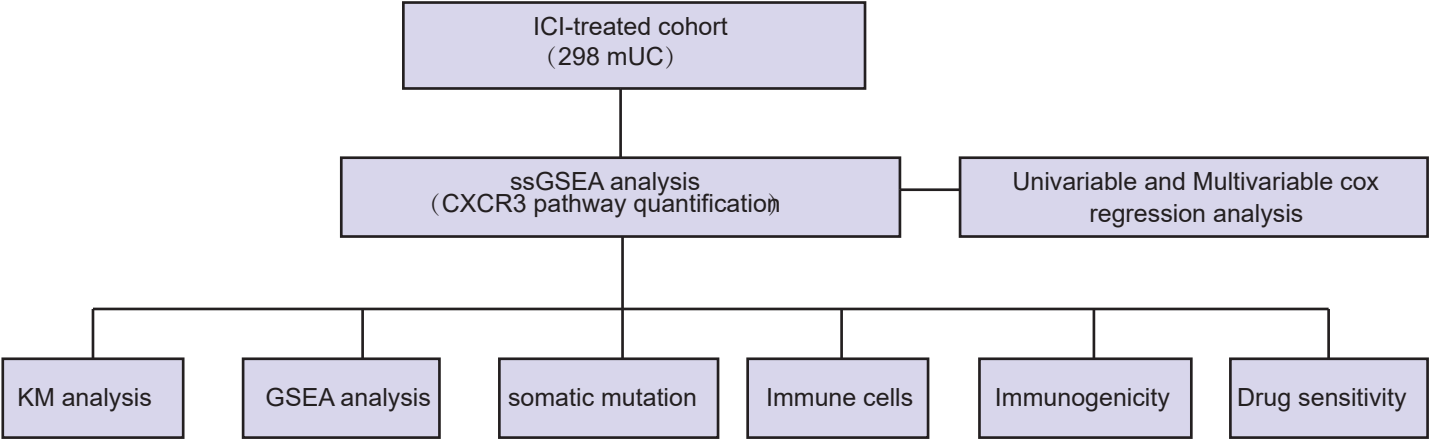

B

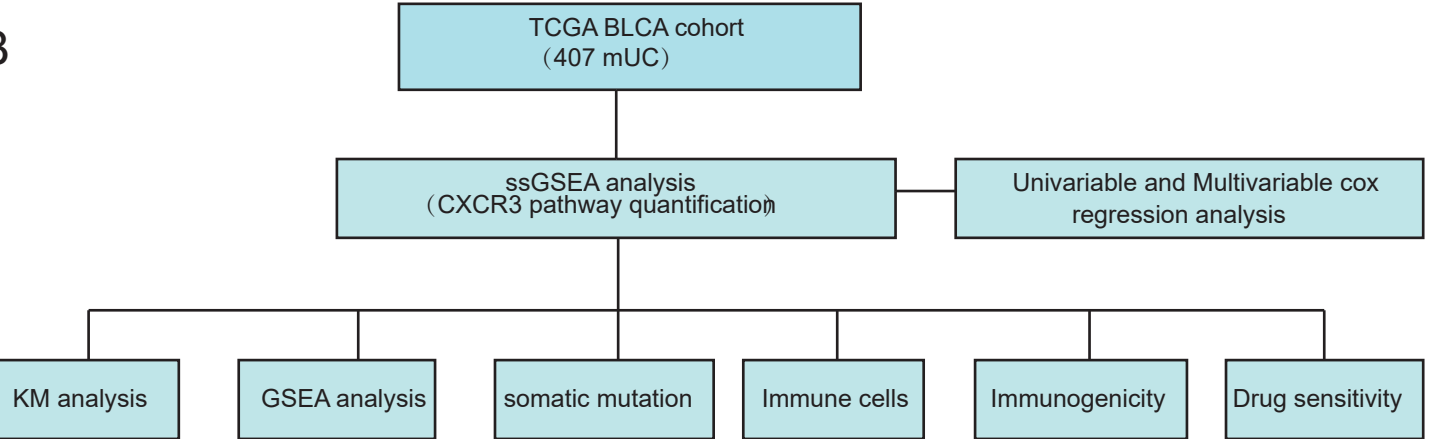

C

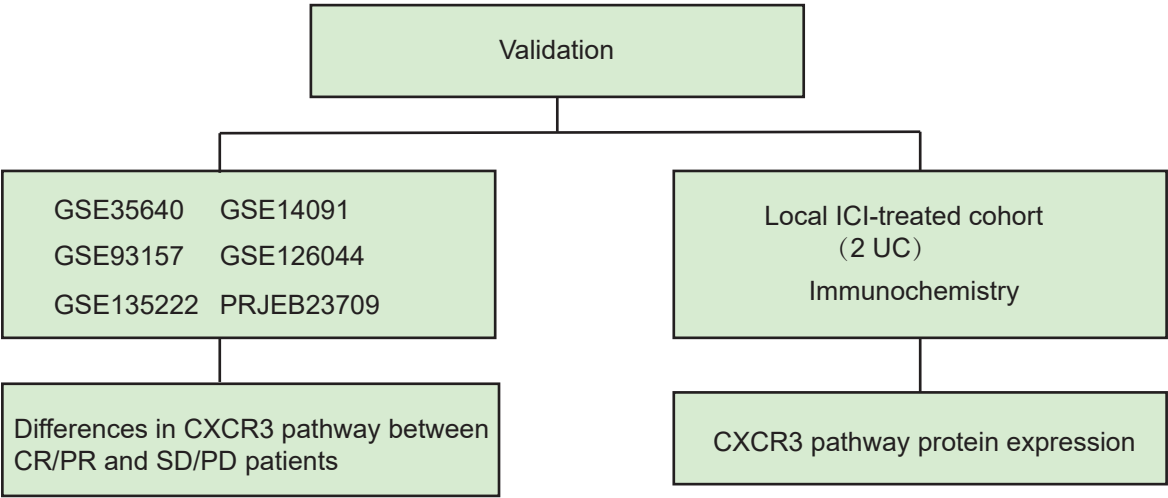

Supplement: Supplementary file 1 — Additional file 1: Figure S1. A–C Flow chart of the data processing of this study. [file 12935_2022_2604_MOESM1_ESM.pdf]

Response  
Nonresponse

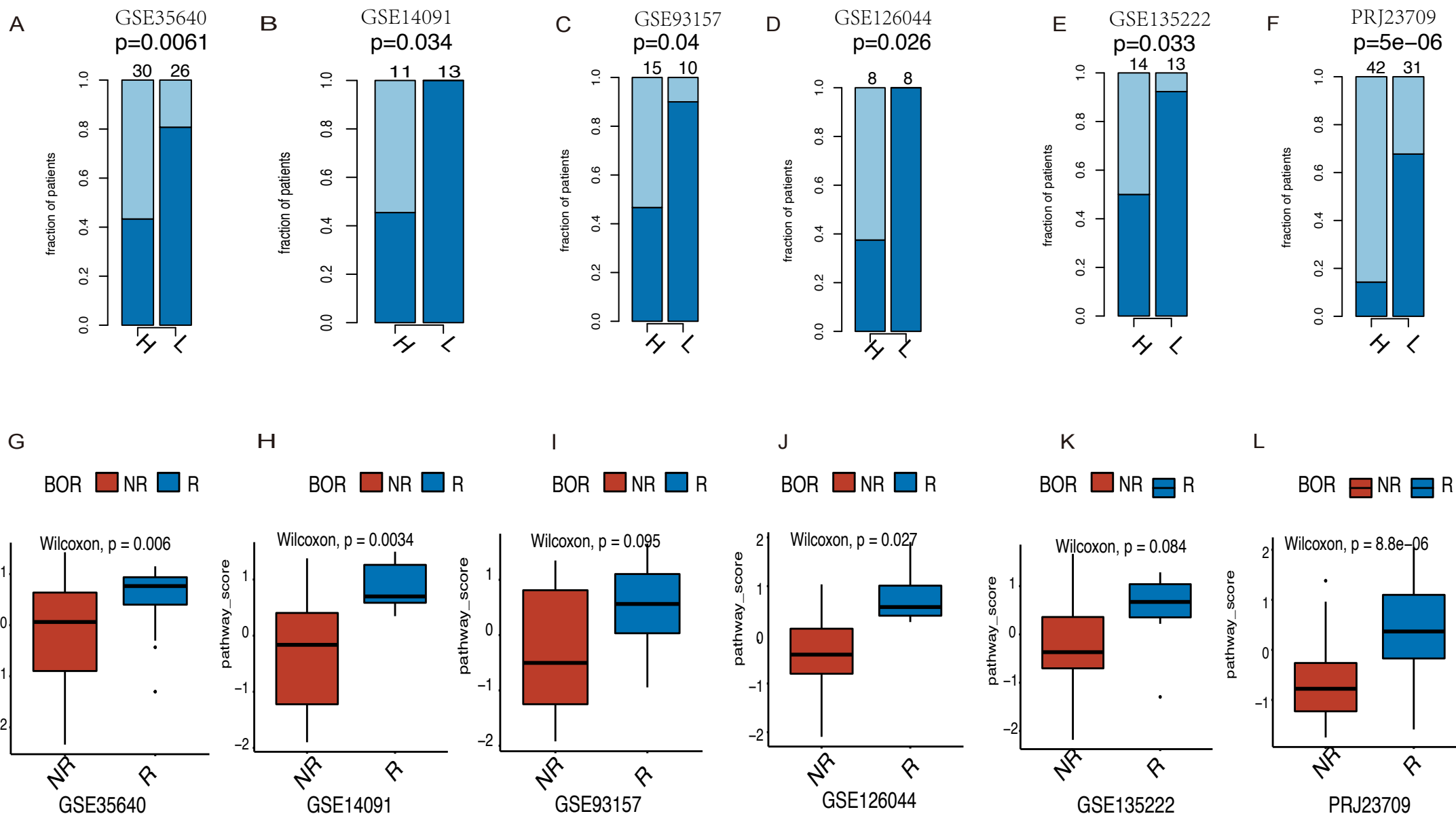

Supplement: Supplementary file 2 — Additional file 2: Figure S2. Validation of relationship between CXCR3 pathway activation and efficacy of ICIs in cancer patients. A–F The differences in the proportions of mUC patients with different responses to ICIs between CXCR3-high and CXCR3-low patients in the other six ICI cohorts. Light blue color represents the responders; dark blue color represents the non-responders. G–L The differences in activation of the CXCR3 pathway in CXCR3-high and CXCR3-low patients from the other six ICI cohorts. NR no response, R response. [file 12935_2022_2604_MOESM2_ESM.pdf]

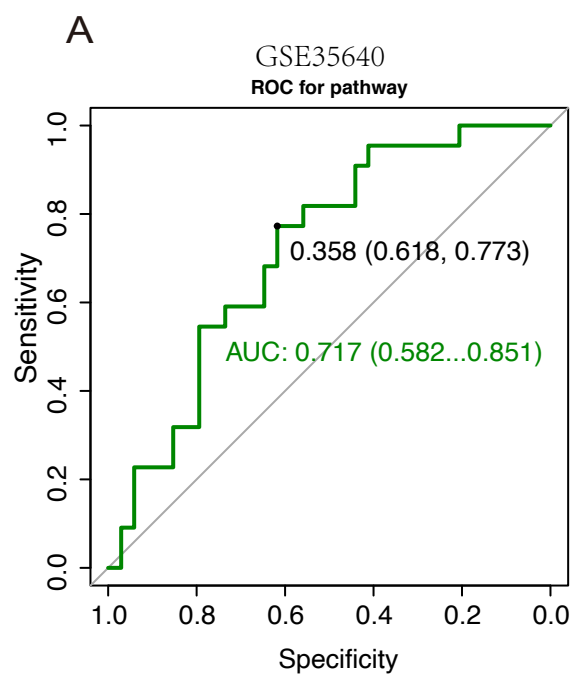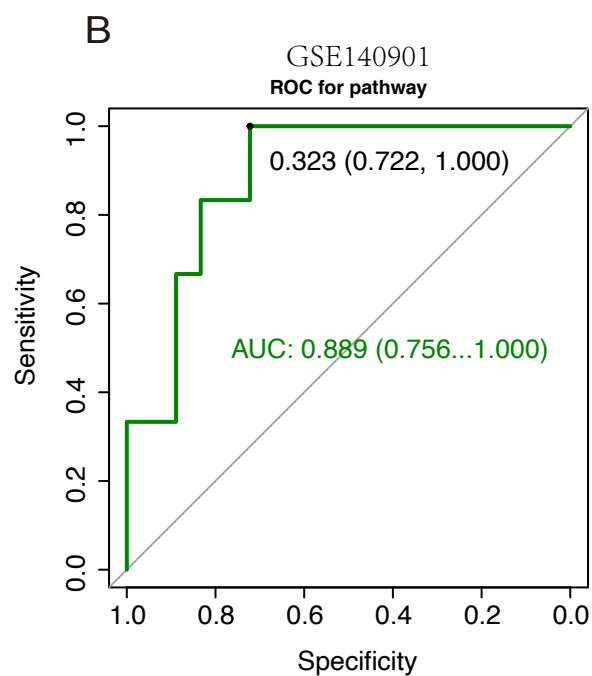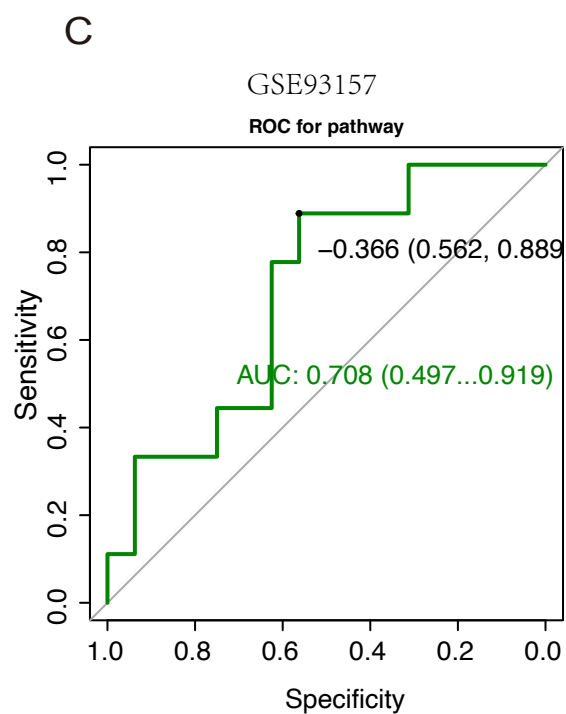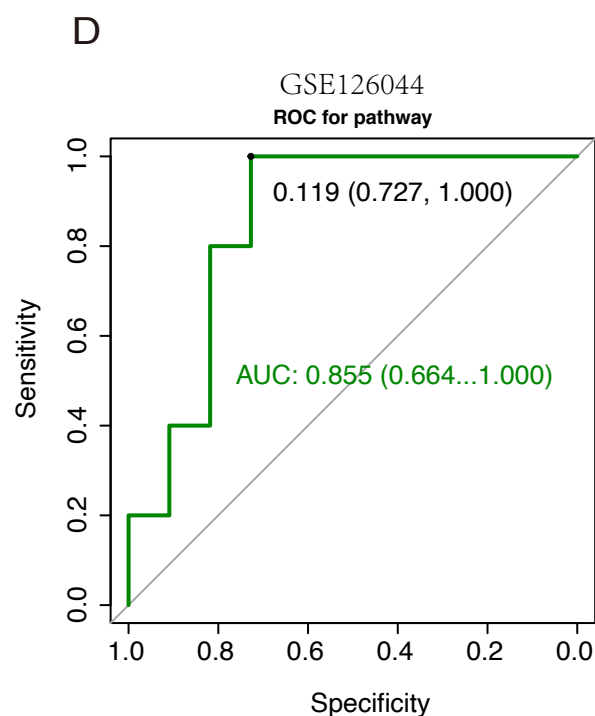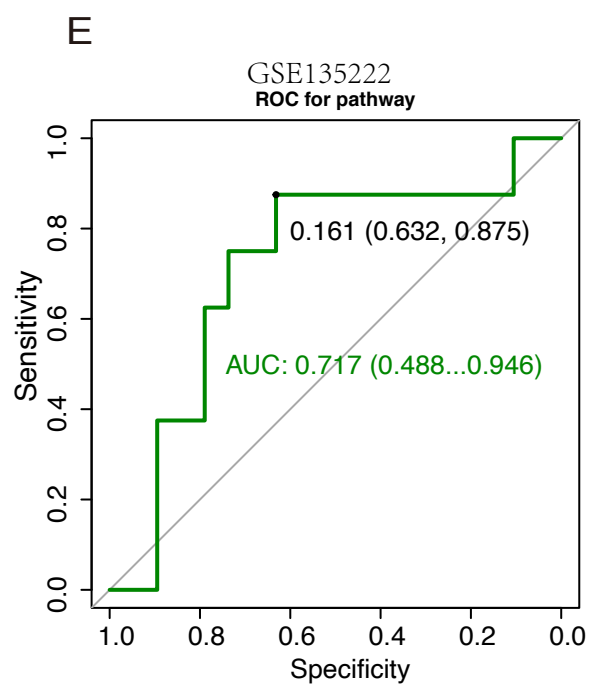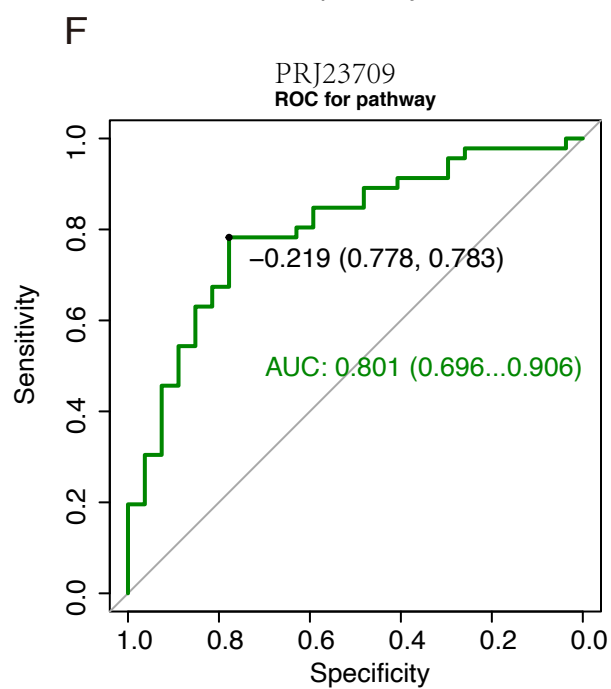

Supplement: Supplementary file 3 — Additional file 3: Figure S3. A–F Receiver operating characteristic (ROC) curve for the predictive value of ssGSEA scores of CXCR3 pathway in the other six validation cohorts. [file 12935_2022_2604_MOESM3_ESM.pdf]

A

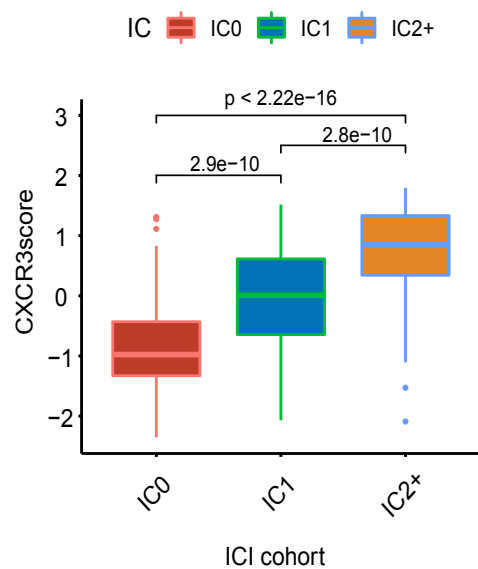

B

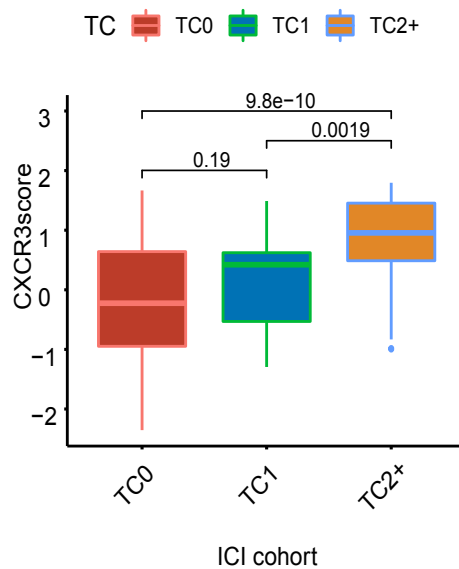

C

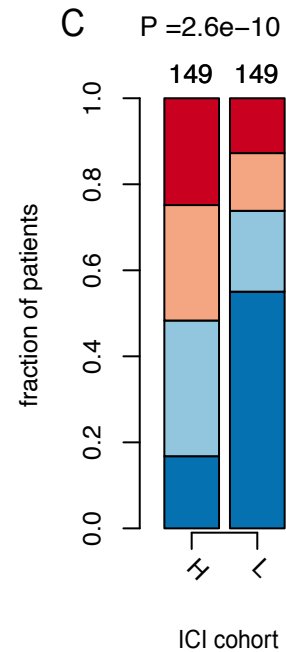

D

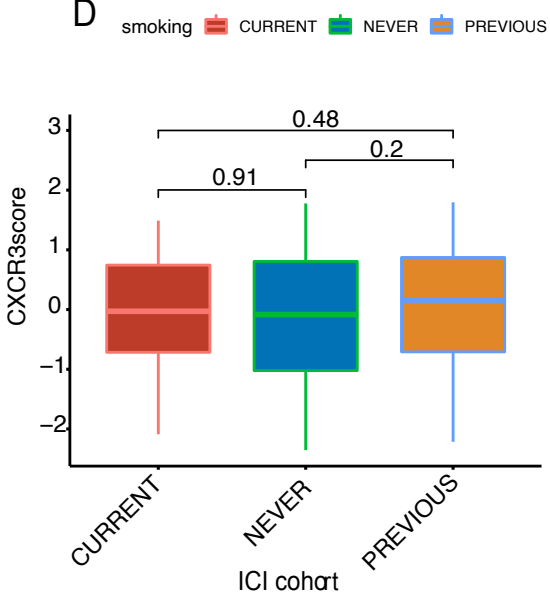

E

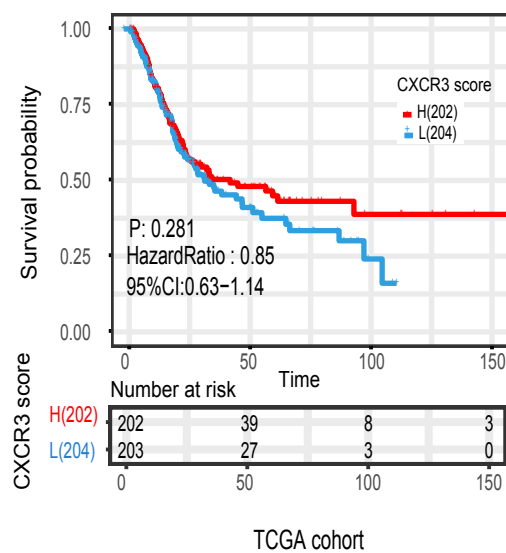

F

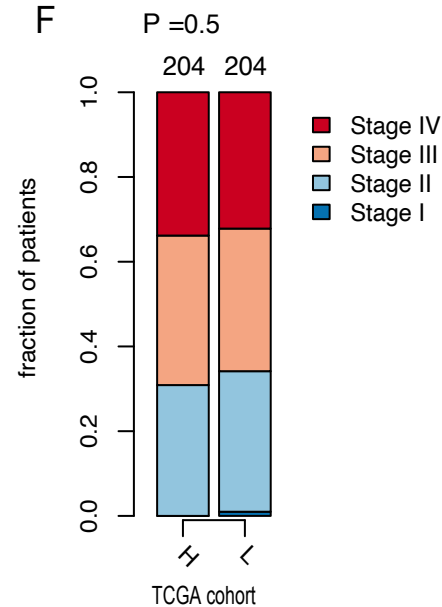

G

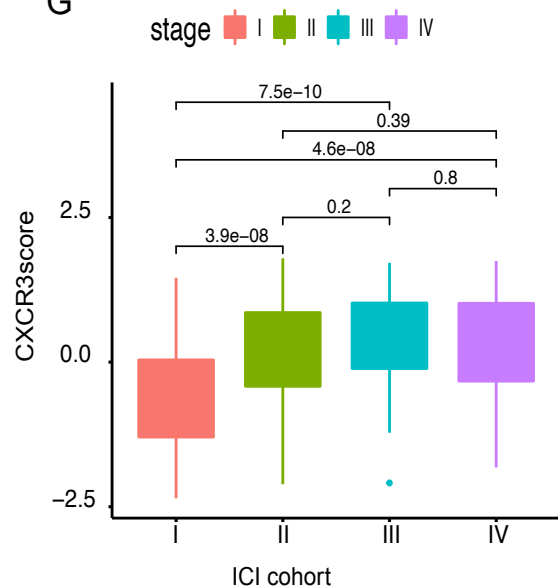

H

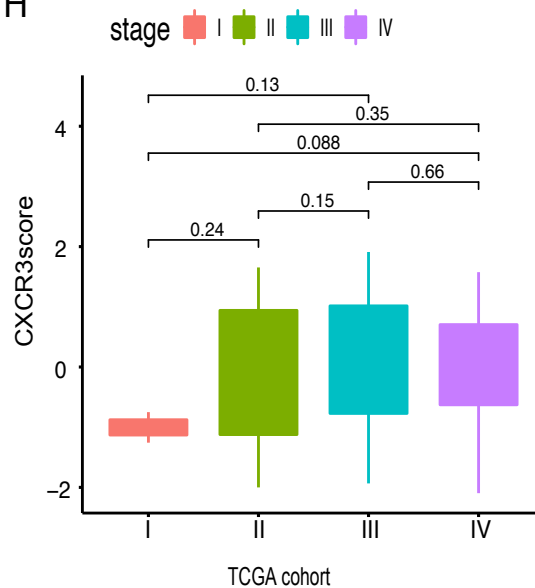

Supplement: Supplementary file 4 — Additional file 4: Figure S4. A Differences in CXCR3 pathway activation between patients with different immune cells’PD-L1 expression in the ICI cohort. B Differences in CXCR3 pathway activation between patients with different tumor cells’PD-L1 expression in the ICI cohort. C Differences in tumor stages in patients with different levels of CXCR3 pathway activation in the ICI cohort. D Differences in patients’ smoking history for different levels of CXCR3 pathway activation in the ICI cohort. E Kaplan-Meier survival curves for OS in CXCR3-high (n =202) and CXCR3-low (n =204) patients in TCGA BLCA cohort. F Differences in tumor stages in patients with different levels of CXCR3 pathway activation in the TCGA BLCA cohort. G Differences in CXCR3 pathway activation levels between patients with different tumor stages in ICI cohort. H Differences in CXCR3 pathway activation levels between patients with different tumor stages in TCGA BLCA cohort. [file 12935_2022_2604_MOESM4_ESM.pdf]

# TCGA cohort

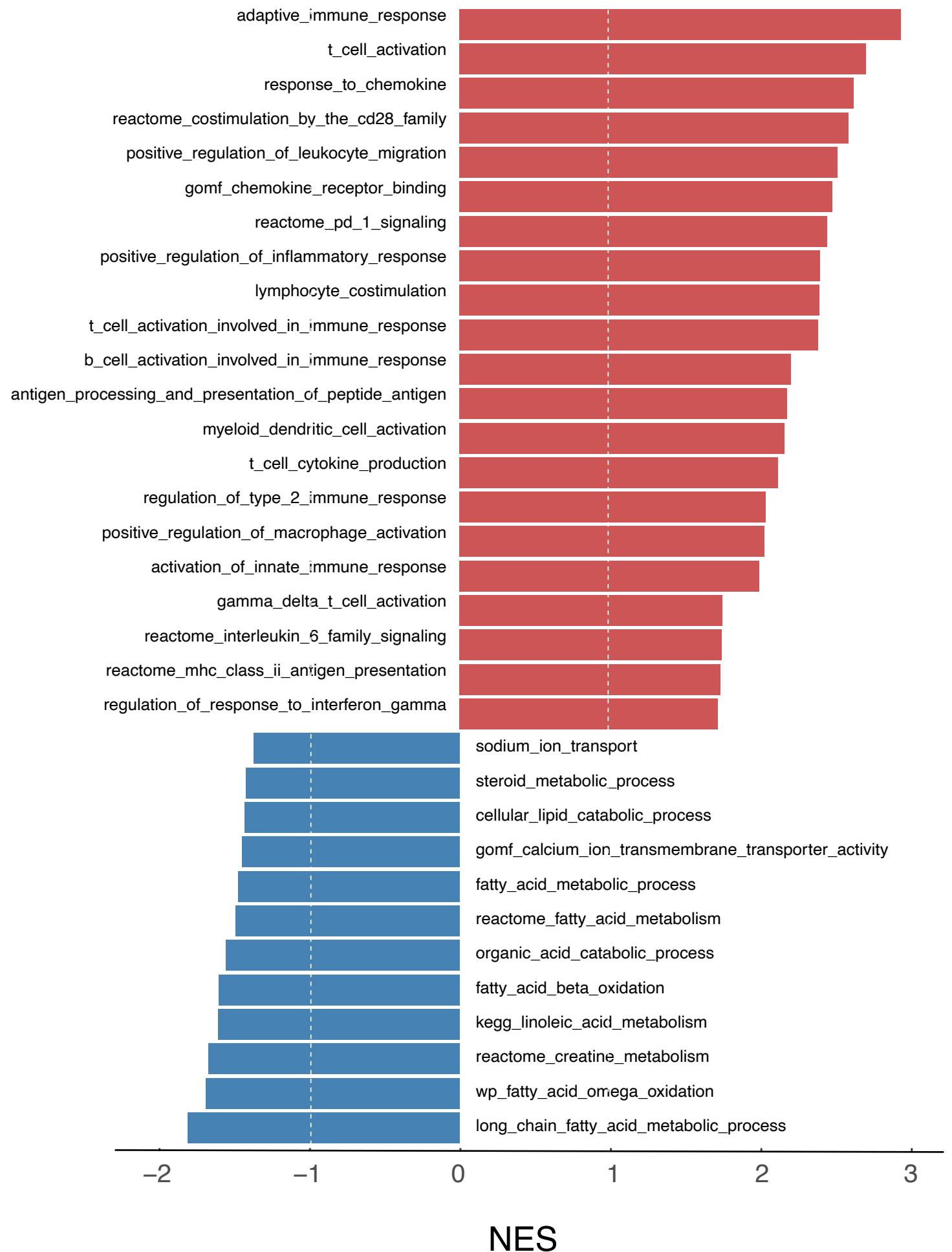

Supplement: Supplementary file 5 — Additional file 5: Figure S5. Histogram showing that the ssGSEA score of the immune-related and lipid metabolism-related signaling pathways set was different in CXCR3-high and CXCR3-low patients from TCGA BLCA cohort (logFC < 0, p < 0.05). ES > 0 means that the corresponding pathway is significantly enriched in CXCR3-high patients, while ES <0 means the opposite. [file 12935_2022_2604_MOESM5_ESM.pdf]

# ICI cohort

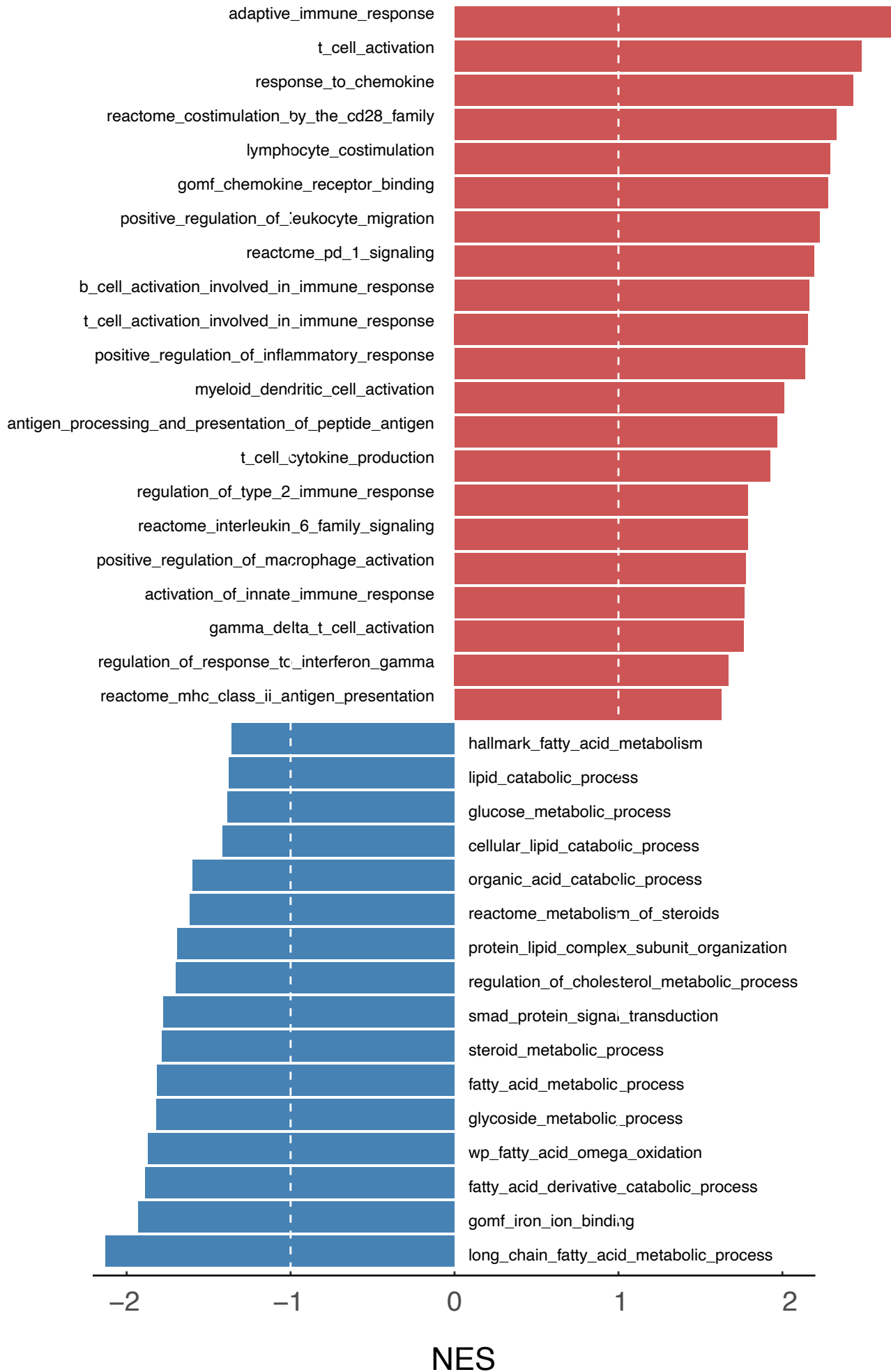

Supplement: Supplementary file 6 — Additional file 6: Figure S6. Histogram showing that the ssGSEA score of the immune-related and lipid metabolism-related signaling pathways set was different in CXCR3-high and CXCR3-low patients from ICI cohort (logFC < 0, p < 0.05). ES > 0 means that the corresponding pathway is significantly enriched in CXCR3-high patients while ES < 0 means the opposite. [file 12935_2022_2604_MOESM6_ESM.pdf]
